# Smoking Habit and *Interleukin 1B C-31T* Polymorphism

**DOI:** 10.2188/jea.11.120

**Published:** 2007-11-30

**Authors:** Nobuyuki Hamajima, Nobuyuki Katsuda, Keitaro Matsuo, Toshiko Saito, Lucy Sayuri Ito, Masahiko Ando, Manami Inoue, Toshiro Takezaki, Kazuo Tajima

**Affiliations:** 1 Division of Epidemiology and Prevention, Aichi Cancer Center Research Institute.; 2 Department of Health, Nagoya Municipal Government.; 3 Nagoya University Graduate School of Medicine.; 4 Department of Preventive Medicine, Nagoya University Graduate School of Medicine.

**Keywords:** smoking, genetic factors, *Interleukin 1B* polymorphism

## Abstract

Recent studies suggest that smoking habit may relate to genetic traits. This study examines the association with a polymorphism (*C-31T*) of *interleukin 1B* (*IL-1B*), which encodes IL-1*β*, a multifunctional pro-inflammatory cytokine. Since the *T* allele makes a TATA box, the allele is thought to be responsible for a higher potency of *IL-1B* expression, indicating that individuals harboring the *T* allele are prone to inflammation. The study subjects were two different populations; 241 non-cancer outpatients (118 males and 123 females) at a cancer hospital and 462 examinees (127 males and 335 females) of a health checkup program provided by a local government. Current smokers were 36.4% for the male outpatients, 9.8% for the female outpatients, 38.6% for the male examinees, and 5.6% for the female examinees. The sex-age-adjusted odds ratios of current smokers were calculated for the genotypes with the *T* allele relative to the *CC* genotype by an unconditional logistic model. The estimate was 0.45 (95% confidence interval, 0.21-0.97) for the outpatients, and 0.83 (0.42-1.61) for the examinees. Although not significant for the examinees, the observed associations suggest that this polymorphism may influence smoking behavior through an inflammatory response of the respiratory tract to cigarette smoke.

## MATERIALS AND METHODS

Smoking habit is maintained by psychological and/or physical addiction. Recent studies suggest that both aspects of the addiction may be partly explained by genetic polymorphisms relating to neurotransmitters and nicotine metabolism^1^^)^. There are two neurotransmitters, dopamine and serotonin, which relate to a psychological reward. To date, the polymorphisms of dopamine receptor genes (*DRD2*^2^^-^^4^^)^ and *DRD4*^5^^)^), dopamine transporter gene (*SLC6A3*)^6^^, ^^7^^)^, and serotonin transporter gene (*5-HTT*)^8^^)^ have been reported to be associated with smoking habit, although inconsistent results have been also reported^9^^-^^11^^)^. The number of cigarettes was also reported to be associated with these polymorphisms^12^^)^. Nicotine is metabolized into cotinine by cytochrome p450 2A6 (*CYP2A6*). The reduced enzyme activity resulting in excretion of nicotine in urine was observed for individuals with entire *CYP2A6* gene deleted^13^^)^. The same amount of nicotine inhalation means a stronger effect for smokers with the genotype than for those with other genotypes, which may prevent to become nicotine dependent^14^^)^.

Another mechanism, an inflammation-prone constitution may relate to smoking habit. Inflammation of the respiratory tract by cigarette smoke evokes an unpleasant feeling, which may make persons to keeping away from smoking consciously or unconsciously. Interleukin 1*β* (IL-1*β*) is a pro-inflammatory cytokine, which triggers a cascade of inflammation reaction through the induction of inflammation-related substances including iNOS and TNF-*α*^15^^)^. IL-1ra, the receptor antagonist for IL-1*β*, binds IL-1 receptor type I competitively, resulting in the inhibition of the IL-1*β* activity^15^^)^. Interleukin-1s have been reported to play a role in several respiratory diseases^16^^-^^22^^)^. The gene *IL-1B* encoding IL-1*β* has three *C*-to-*T* polymorphisms at -511, -31, and 3954 base pairs from the transcriptional start site. Among them, the polymorphism *C-31T* is though to be associated with the gene expression; the *T* allele making a TATA box, a promoter sequence^23^^)^. The serum level of IL -1*β* was reported in Europe to be higher for individuals harboring the *T* allele of *C-511T*^24^^)^, which is very tightly linked with the *T* allele of *C-31T* among Caucasians^23^^)^. In this study, the association between smoking habit and a functional polymorphisms of IL-1B *C-31T* was examined for two Japanese populations; outpatients and a health checkup examinees. To our knowledge, this is the first report on the association in the world. The IL-1B polymorphism was the second polymorphism examined with smoking for the outpatients, and the first for the examinees.

## MATERIALS AND METHODS

### Study subjects

The subjects were sampled from the outpatients who visited Aichi Cancer Center Hospital in 1999, and from the examinees who attended a health checkup program supported by the Nagoya municipal government in 2000.

The outpatients were those who underwent gastroscopy and agreed to participate in a *Helicobacter pylori* eradication program. A written informed consent was obtained for gene polymorphism tests without specification of names of polymorphims, and for lifestyle questionnaire including smoking habit. For the eradication program, 283 outpatients (138 males and 145 females) were enrolled. Excluding 42 participants (38 with a history of cancer, 3 hepatitis virus carriers whose blood was not stored, and 1 who refused blood sampling after entry), remaining 241 outpatients (118 males and 123 females) were used for the present analysis. They included 97 (40.2% out of 241) participants stated to be under medication for 107 diseases (not confirmed by their medical records); 23 with gastric/duodenal ulcer, another 23 for so-called gastritis, 16 with hypertension, 8 for pain including arthritis and lumbago, 7 with diabetes mellitus, 7 with hyperlipidemia, 3 with ischmic heart disease, 3 with thyroid disease, 2 with gynecological disease, 2 with hyperuricaemia, 2 Meniere disease, 2 with prostate disease, 1 with ulcerative colitis, 1 with pancreatitis, 1 with asthma, 1 with arrhythmia, 1 for epilepsy, 1 for neurosis, 1 with liver cirrhosis, 1 for ulticaria, and 1 after cerebral infarction.

The examinees were inhabitants of West ward of Nagoya City. A written informed consent to anonymous use of the residual blood as well as information on demographic characteristics and smoking was obtained after the blood draw for the health checkup. Usually, about 2 ml of blood was left after the routine tests. No extra blood draw was conducted. Out of 489 examinees invited to the study, 468 (95.7%) agreed to provide their residual blood for genetic tests and related information. Three residual blood samples were not available for DNA extraction. Genotyping of *IL-1B* *C-31T* did not succeed for three samples. The remaining 462 examinees were the subjects of this study.

### Genotyping

DNA was extracted from 200 *μ*l buffy coat preserved at -40°C by QIAamp DNA Blood Mini Kit (QIAGEN Inc., Valencia, CA). The genotyping was conducted by a novel PCR technique, PCR-CTPP (polymerase chain reaction with confronting two-pair primers)^25^^)^, as described in another paper^26^^)^. The present study had been approved by Ethical Committee at Aichi Cancer Center before the study started (Ethical Committee Approval Numbers 12-23 and 11-12)

### Statistical analysis

An unconditional logistic model was applied for estimating odds ratios (ORs) by a computer program STATA Version 6 (STATA Corporation, College Station, TX). Age-adjustment for the ORs was conducted as a continuous variable. The fitness for Hardy-Weinberg equilibrium was examined also by STATA.

## RESULTS

Table 1 shows the sex and age distributions of the subjects according to smoking status. The age ranged from 39 to 69 years for the outpatients and 32 to 85 years for the examinees. Current smokers were 36.4% in males and 9.8% in females among the outpatients at Aichi Cancer Center Hospital, and 38.6% in males and 5.6% in females among the examinees. The number of cigarettes per day ranged 1 to 60 (76.4% for current smokers with 20 cigarettes or more) for the outpatients, and 1 to 50 (55.2% for current smokers with 20 cigarettes or more) for the examinees. A 18.2% of 55 outpatients and 22.3% of 67 examinees stated that they had started smoking at age less than 20. Among the male outpatients, 41 (34.7% of 118) answered to quit smoking before their visits, while the corresponding value was 4.1% for the female outpatients, 5.5% for the male examinees, and 1.5% for the female examinees. All of the former smokers stated that they had quit smoking before one year.

**Table 1.** Sex and age distributions of the subjects according to smoking status.

Age	Males				Females			
	Never	Former	Current	Total	Never	Former	Current	Total
**Outpatients at Aichi Cancer Center Hospital**								
39 - 49	5	7	11	23	15	0	8	23
(%)	(21.7)	(30.4)	(47.8)	(100)	(65.2)	(0.0)	(34.8)	(100)
50 - 59	11	12	11	34	49	4	3	56
(%)	(32.4)	(35.3)	(32.4)	(100)	(87.5)	(7.1)	(5.4)	(100)
60 - 69	18	22	21	61	42	1	1	44
(%)	(29.5)	(36.1)	(34.4)	(100)	(95.5)	(2.3)	(2.3)	(100)

Total	34	41	43	118	106	5	12	123
(%)	(28.8)	(34.7)	(36.4)	(100)	(86.2)	(4.1)	(9.8)	(100)

**Examinees who attended a health checkup program**								
32 - 39	2	0	7	9	36	1	3	40
(%)	(22.2)	(0.0)	(77.8)	(100)	(90.0)	(2.5)	(7.5)	(100)
40 - 49	3	0	4	7	42	1	8	51
(%)	(42.9)	(0.0)	(57.1)	(100)	(82.4)	(2.0)	(15.7)	(100)
50 - 59	6	0	4	10	87	3	5	95
(%)	(60.0)	(0.0)	(40.0)	(100)	(91.6)	(3.2)	(5.3)	(100)
60 - 69	39	4	22	65	114	0	1	115
(%)	(60.0)	(6.2)	(33.8)	(100)	(99.1)	(0.0)	(0.9)	(100)
70 - 79	18	2	12	32	29	0	1	30
(%)	(56.3)	(6.3)	(37.5)	(100)	(96.7)	(0.0)	(3.3)	(100)
80 - 85	3	1	0	4	4	0	0	4
(%)	(75.0)	(25.0)	(0.0)	(100)	(100.0)	(0.0)	(0.0)	(100)

Total	71	7	49	127	312	5	18	335
(%)	(55.9)	(5.5)	(38.6)	(100)	(92.9)	(1.5)	(5.6)	(100)

**Table 2.** *IL-1B C-31T* genotype distributions according to smoking status.

	Males				Females			
	Never	Former	Current	Total	Never	Former	Current	Total
**Outpatients at Aichi Cancer Center Hospital**								
*CC*	4	7	13	24	14	1	3	18
(%)	(16.7)	(29.2)	(54.2)	(100)	(77.8)	(5.6)	(16.7)	(100)
*CT*	22	27	21	70	56	1	6	63
(%)	(31.4)	(36.6)	(30.0)	(100)	(88.9)	(1.6)	(9.5)	(100)
*TT*	8	7	9	24	36	3	3	42
(%)	(33.3)	(29.2)	(37.5)	(100)	(85.7)	(7.1)	(7.1)	(100)

Total	34	41	43	118	106	5	12	123
(%)	(28.8)	(34.7)	(36.4)	(100)	(86.2)	(4.1)	(9.8)	(100)

**Examinees who attended a health checkup program**								
*CC*	22	0	15	37	71	3	5	79
(%)	(59.5)	(0.0)	(40.5)	(100)	(89.9)	(3.8)	(6.3)	(100)
*CT*	24	3	18	45	125	2	11	138
(%)	(53.3)	(53.3)	(40.0)	(100)	(90.6)	(1.4)	(8.0)	(100)
*TT*	25	4	16	45	116	0	2	118
(%)	(55.6)	(8.9)	(35.6)	(100)	(98.3)	(0.0)	(1.7)	(100)

Total	71	7	49	127	312	5	18	335
(%)	(55.9)	(5.5)	(38.6)	(100)	(92.9)	(1.5)	(5.6)	(100)

When both sexes were combined, the genotype of *IL-1B* *C-31T* was 17.4% for the *CC* genotype, 55.2% for the *CT* genotype, and 27.4% for the *TT* genotype among the outpatients, and 25.1%, 39.6%, and 35.3% among the examinees, respectively. The genotype distribution for the outpatients was in Hardy-Weinberg equilibrium (*χ*2=3.18, p=0.075), but not for the examinees (*χ*2=18.39, p<0.00001).

The male smokers were significantly more (*χ*2=4.09, p<0.05) among the outpatients with the *CC* genotype (54.2%, 13/24) than among those with the other genotypes (31.9%, 30/94). Though not significant, the similar tendency was observed for the female outpatients. Concerning the examinees, the *CT* genotype as well as the *CC* genotype tended to be smokers compared with the *TT* genotype.

Table 3 shows the ORs of being current smokers for each population. The outpatients with the *CT* or *TT* genotype were significantly reduced risk of being current smokers compared with those with the CC genotype; the sex-age-adjusted OR was 0.45 (95% confidence interval, 0.21-0.97). The reduction of the adjusted OR was less clear for the examinees; adjusted OR=0.83 (0.43-1.61). When the OR was calculated for the ever smokers (current smokers and former smokers), similar results were obtained for the outpatients (Table 4). Concerning the examinees, the OR was significant for the females with the *TT* genotype; the crude OR=0.15 (0.23-1.36), and the adjusted OR=0.13 (0.03-0.64).

**Table 3.** Odds ratios (ORs) and 95% confidence intervals (95%CIs) of being current smokers.

	Outpatients				Examinees			
	cOR^a^	(95%CI)	aOR^b^	(95%CI)	cOR^a^	(95%CI)	aOR^b^	(95%CI)
Males								
*CC*	1	(Reference)	1	(Reference)	1	(Reference)	1	(Reference)
*CT*	0.36	(0.14 - 0.94)	0.37	(0.14 - 0.97)	0.98	(0.40 - 2.37)	1.01	(0.40 - 2.50)
*TT*	0.51	(0.16 - 1.61)	0.51	(0.16 - 1.64)	0.81	(0.33 - 1.98)	0.83	(0.33 - 2.10)
*CT/TT*	0.40	(0.16 - 0.99)	0.41	(0.16 - 1.01)	0.89	(0.41 - 1.95)	0.92	(0.41 - 2.06)
Females								
*CC*	1	(Reference)	1	(Reference)	1	(Reference)	1	(Reference)
*CT*	0.53	(0.12 - 2.35)	0.85	(0.17 - 4.30)	1.28	(0.43 - 3.83)	1.09	(0.36 - 3.34)
*TT*	0.38	(0.07 - 2.12)	0.34	(0.05 - 2.14)	0.26	(0.05 - 1.35)	0.22	(0.04 - 1.19)
*CT/TT*	0.47	(0.11 - 1.93)	0.58	(0.13 - 2.62)	0.79	(0.27 - 2.29)	0.68	(0.23 - 2.01)
Combined								
*CC*	1	(Reference)	1	(Reference)	1	(Reference)	1	(Reference)
*CT*	0.41	(0.20 - 0.88)	0.44	(0.19 - 0.99)	0.90	(0.48 - 1.69)	1.09	(0.53 - 2.24)
*TT*	0.36	(0.15 - 0.87)	0.47	(0.18 - 1.21)	0.60	(0.30 - 1.18)	0.59	(0.27 - 1.30)
*CT/TT*	0.40	(0.19 - 0.81)	0.45	(0.21 - 0.97)	0.75	(0.43 - 1.34)	0.83	(0.43 - 1.61)

^a^ Crude OR.

^b^ Age-adjusted OR for each sex, and sex-age-adjusted OR for both sex combined.

**Table 4.** Odds ratios (ORs) and 95% confidence intervals (95%CIs) of being ever smokers.

	Outpatients				Examinees			
	cOR^a^	(95%CI)	aOR^b^	(95%CI)	cOR^a^	(95%CI)	aOR^b^	(95%CI)
Males								
*CC*	1	(Reference)	1	(Reference)	1	(Reference)	1	(Reference)
*CT*	0.44	(0.13 - 1.43)	0.45	(0.14 - 1.47)	1.28	(0.53 - 3.09)	1.32	(0.54 - 3.21)
*TT*	0.40	(0.10 - 1.57)	0.40	(0.10 - 1.59)	1.17	(0.49 - 2.83)	1.21	(0.50 - 2.97)
*CT/TT*	0.43	(0.13 - 1.36)	0.44	(0.14 - 1.39)	1.23	(0.56 - 2.67)	1.26	(0.58 - 2.78)
Females								
*CC*	1	(Reference)	1	(Reference)	1	(Reference)	1	(Reference)
*CT*	0.44	(0.11 - 1.71)	0.62	(0.15 - 2.58)	0.92	(0.37 - 2.33)	0.77	(0.29 - 2.00)
*TT*	0.58	(0.14 - 2.38)	0.60	(0.14 - 2.62)	0.15	(0.03 - 0.74)	0.13	(0.03 - 0.64)
*CT/TT*	0.49	(0.14 - 1.73)	0.61	(0.17 - 2.26)	0.55	(0.23 - 1.36)	0.46	(0.18 - 1.17)
Combined								
*CC*	1	(Reference)	1	(Reference)	1	(Reference)	1	(Reference)
*CT*	0.53	(0.26 - 1.07)	0.55	(0.27 - 1.12)	0.92	(0.51 - 1.66)	0.92	(0.51 - 1.66)
*IT*	0.38	(0.17 - 0.83)	0.38	(0.17 - 0.84)	0.63	(0.33 - 1.20)	0.63	(0.33 - 1.20)
*CT/TT*	0.47	(0.24 - 0.93)	0.49	(0.25 - 0.96	0.78	(0.46 - 1.34)	0.77	(0.45 - 1.33)

^a^ Crude OR.

^b^ Age-adjusted OR for each sex, and sex-age-adjusted OR for both sex combined.

## DISCUSSION

Epidemiologically, the observed association was unlikely to be introduced by information bias, selection bias, and confounding. The genotyping was conducted, masking their smoking status. The subjects did not know their genotype. Sampling was conducted before getting the information on smoking status and genotype. The cases (smokers) and controls (non-smokers) were recruited in the same framework. Possible confounding factors, age and sex, were adjusted by the logistic model. Generally speaking, genotype distribution is independent on age and sex, so both factors are not confounders. We listed in this paper the crude ORs as well as the adjusted ORs. The effect of population stratification causing the confounding seems very limited in Japan, in comparison with the multiethnic countries such as the United States.

Although it is intuitively realized that persons sensitive to tobacco smoke cannot be smokers, there are no biological and/or epidemiologic studies which examine the association between the sensitivity and smoking behavior. It was reported that cigarette smoke extracts suppress the *in vitro* production of IL-1*β*, as well as IL-2, interferon (IFN) - *γ* and tumor necrosis factor (TNF) - *α*^27^^)^, which was considered to relate to a localized suppression of immune responses in the lungs^28^^)^. Meanwhile, smoking increases the incidence and severity of respiratory tract infections,^29^^)^ suggesting that cigarette smoke tends to induce the inflammation of the respiratory tract. Of interest is a report that IL-1*β* release by cigarette smoke exposure was more from bronchial epithelial cell cultures of the never-smokers than from those of the smokers^30^^)^. The biological finding is consistent with our results observed for the outpatients; the individuals with a lower level of IL-1*β* production are likely to become smokers.

The polymorphisms of *IL-1A* *C-889T* and *IL-1RN* 86-bp variable number of tandem repeat reportedly related to the serum IL-1*β* level^24^^,^^31^^)^. Among Japanese, the CC genotype of *IL-1A* (83.4%, n=241) and homozygous genotype of 4-repeat allele (90.0%, n=241) were dominant^26^^)^ so that the effect of the genotype combination was difficult to be examined in this study. Genetic polymorphisms have been also reported for IL-1 receptors type I and type II which may modify the effects of IL-1*β*^15^^)^, but the genotype frequencies have not examined for Japanese.

The allelic distribition of *IL-1B* *C-31T* polymorphism was not in Hardy-Weinberg equilibrium for the examinees of this study, probably due to the non-random participation in the health checkup program. The participation with his/her family members was not rare, though estimating the proportion of such participants was not possible because the identification was deleted in the process to make the samples anonymous.

The association with the *IL-1B* polymorphism was stronger for the outpatients than for the examinees. An effect modifier may exist for the association. All the outpatients were ones who visited digestive disease clinics and underwent endoscopy. They consisted of the individuals with upper abdominal symptoms and non-symptomatic individuals who advised to visit Aichi Cancer Center or who visited the Hospital for the purpose of the annual endoscopy checkup. The latter were the majority of the outpatients, but the percentage of the individuals with symptoms was thought to be naturally higher for the outpatient population than for the examinees population. The higher percentage of the former smokers among the male outpatients may also indicate the modifier underlying the different association strength.

The modern epidemiology is based on a multifactorial model, indicating that a risk factor plays a role for a subgroup of population. When the subgroup accounts for a smaller proportion in the study subjects, the estimated relative risk is diluted to a larger degree. The observed difference in the association with the *IL-1B* polymorphism between the outpatients and the examinees suggested that other factors on genetic traits and/or lifestyle affect the association with the *IL-1B* polymorphism . The proportion of subjects influenced by the underlying other factors might be larger in the outpatient population than in the examinee population.

There are many studies on polymorphisms which report inconsistent results from different subjects. Two reasons for the inconsistency are considered; different linkage with a truly responsible gene and modification by gene-gene interactions and/or gene-environment interactions. In this study, the ethnicity of the subjects was the same, so the different linkage seemed unlikely. Although the different strength of the association with *IL-1B* *C-31T* between the two subjects from different sources could be caused by a random effect, it was also likely that some factors modified the association. Further studies on the modifiers are required.
